# Are Mathematical and Musical Abilities Related Beyond Intelligence?

**DOI:** 10.3390/jintelligence14030039

**Published:** 2026-03-02

**Authors:** Michaela A. Meier, Lara Spitzley, Serra Ulusoy, Alexandra Hubmann, Rylie DelaCruz, Roland H. Grabner, Daniel Müllensiefen

**Affiliations:** 1 Department of Psychology, University of Graz, 8010 Graz, Austria; lara.spitzley@outlook.com (L.S.); roland.grabner@uni-graz.at (R.H.G.); 2 Department of Psychology, Goldsmiths, University of London, London SE14 6NW, UK; serraulusoy@gmail.com (S.U.); daniel.muellensiefen@uni-hamburg.de (D.M.); 3 Institute for Systematic Musicology, University of Hamburg, 20354 Hamburg, Germany

**Keywords:** mathematics, music, intelligence, individual differences, expertise

## Abstract

Numerous studies have aimed to improve mathematical achievement via musical interventions because it is argued that music and mathematics draw on related representations and similar skills. However, findings on their effectiveness are inconclusive. This might be because studies neglect to investigate the cognitive mechanisms that might link musical and mathematical abilities. Therefore, this study aimed to systematically investigate the relationships between facets of musical and mathematical ability while taking into account intelligence as a possible explanation for this link. Among 170 young adults with backgrounds in mathematics and/or music, as well as control subjects, we measured mathematical abilities using basic numerical abilities, arithmetic fluency, and higher mathematical competencies. Musical abilities were assessed using beat alignment, mistuning perception, and melodic discrimination. Intelligence was assessed using a verbal, figural, and numerical scale of an intelligence test. Using a latent variable model, we found a moderate to strong positive association between mathematical and musical abilities. However, after including intelligence as a predictor for both mathematical and musical abilities in our model, the relationship between mathematics and music became nonsignificant. These results imply that intelligence accounts for a substantial proportion of the association between mathematical and musical abilities.

## 1. Introduction

It has often been argued that music and mathematics have a deep relationship (e.g., Vaughn, 2000) because music is based on mathematical principles such as ratios and repeating patterns. As such, musical training can be seen as a promising approach to improving mathematical achievement. However, results are mixed, with some studies reporting small positive effects (e.g., Akın, 2025) and others finding no effect at all (e.g., Sala & Gobet, 2020). In contrast, there is very little empirical evidence on the actual relationship between different facets of musical and mathematical abilities, which should be answered first in this line of research to gain a better understanding of the structural relationships and correspondences between the two domains. Furthermore, a major shortcoming of previous studies is the lack of focus on the cognitive mechanisms that might link musical and mathematical abilities (Vuvan & Sullivan, 2025). Thus, the main aim of this study was to investigate the relationships between facets of musical and mathematical ability, taking into account individual differences in general cognitive ability.

At first glance, mathematics as a purely scientific discipline and music as an expressive art do not seem to have much in common, but a closer look reveals a different picture. From ancient times to the Middle Ages, music and mathematics were both considered to be natural sciences (Papadopoulos, 2002). In antiquity, eminent scholars such as Pythagoras, Plato, and Aristotle already discussed the similarities between the two disciplines. Furthermore, ancient Greek education included four disciplines, called the quadrivium: number theory, geometry, music, and astronomy (Valleriani, 2022), suggesting a close relation. Not only do both mathematics and music use a representational language and a specific symbolic notation, but both are fundamentally concerned with patterns and structures. Musical structures can be described through mathematical concepts such as symmetry, ratio, and discreteness. Further, rhythm, pitch intervals, duration, tempo, and many other musical concepts are naturally measured and represented by numbers. Rhythm, for example, consists of temporal proportions set at mathematical intervals from one another, and pitch intervals are ordered in a system based on number spacing (Shah, 2010). Furthermore, interviews with both lay people (e.g., high school students (Cranmore & Tunks, 2015)) and experts (e.g., mathematicians and mathematics educators, and musicians and music educators (Azaryahu et al., 2024)) showed that many individuals hold the beliefs that mathematics serves as a foundation for music, that mathematical skills influence musical abilities, and that music can be a tool for learning mathematics.

It is not surprising, therefore, that in recent decades, research has taken an educational perspective. Numerous studies have investigated the causal role of musical training on academic achievement in general and mathematics achievement in particular. Researchers argue that engaging in cognitively demanding activities such as music training can foster domain-general cognitive skills, and sometimes it is even claimed to foster general intelligence (Sala & Gobet, 2017). However, empirical findings do not provide clear support for the beneficial effects of music training. Sala and Gobet (2020) reviewed meta-analytic evidence from 54 experimental studies on how music training affects children’s cognitive skills and academic outcomes. While there was an overall small positive effect (*g* = 0.18), this effect disappeared when the quality of the study design was taken into account. Especially those studies that used an active control group and randomized their participants to groups showed homogeneous null or near-zero effects (*g* = −0.02–0.06). Furthermore, this result was comparable for both cognitive skills and academic achievement, so the authors did not report separate effect sizes for the nine studies that included a mathematics outcome. Román-Caballero et al. (2022) argued that non-instrumental music interventions (i.e., interventions not requiring the playing of a musical instrument) have a smaller impact on cognitive skills and academic achievement than music interventions that include the learning of a musical instrument. However, two-thirds of the studies included in Sala and Gobet’s (2020) meta-analysis were non-instrumental interventions. In contrast, in the meta-analyses by Román-Caballero et al. (2022), 32 studies with pre-post designs and at least one control group were examined to assess the effects of learning to play a musical instrument on cognitive skills and academic achievement. Overall, they found a small, positive effect (*g* = 0.26), and in contrast to the previous meta-analysis by Sala and Gobet (2020), they did not find differences in effect size based on whether the study was randomized or non-randomized, or whether the study had an active or passive control group. In addition, Román-Caballero et al. (2022) reported effect sizes separately for each type of cognitive or academic outcome, and the four included studies on mathematics showed a small positive effect (*g* = 0.23), although the effect was not significant (*p* = .316) and the 95% CI [−0.41, 0.87] included both positive and negative effects.

The most recent meta-analysis (Akın, 2025) focused specifically on experimental or quasi-experimental studies that investigated the effect of music interventions on mathematics achievement in K-16 education. Including 55 studies, the results indicated a small to moderate positive effect of music interventions on mathematics achievement (*g* = 0.36). Moderator analyses indicated that effect sizes differed depending on the type of mathematics achievement measured, the instructional mathematics content, and the type of music intervention. The largest effect of musical training was on arithmetic skills (*g* = 0.47), followed by spatial skills and problem solving (both *g* = 0.36) and logical reasoning (*g* = 0.34). This is also supported by the instructional mathematics content, which showed that foundational mathematics content (e.g., numbers, operations, fractions) was more strongly related to music (*g* = 0.46) than higher-level mathematics content (e.g., geometry, linear algebra, integrals; *g* = 0.34). However, according to the authors, the most important finding was the type of music intervention. Here, music–mathematics integrated interventions, which used mathematics and music together in the learning environment, produced the largest effect size (*g* = 0.61) compared to instrumental music interventions (*g* = 0.49) and standardized music interventions (*g* = 0.23).

In addition to these experimental training studies, there are also a few population-based and correlational studies on the relationship between music and mathematics. For example, Guhn et al. (2020) examined population-level educational records of over 112,000 secondary school students in Canada and compared academic outcomes of school music participation to no music participation. Here, students who took school music courses had significantly higher mean exam scores in mathematics (*d* = 0.36), even after controlling for prior academic achievement and socioeconomic status (*d* = 0.25). In contrast, another study (Haimson et al., 2011) examined whether mathematicians have better musical skills. Using a self-report questionnaire assessing musicality (music perception and music memory) and musicianship (music performance and music creation), mathematicians, defined as individuals with a Ph.D. in a mathematical society, did not show higher levels of musical ability compared to literature and language scholars.

However, one major shortcoming of most of these studies is that they do not take individual differences in general cognitive abilities into account at all. For example, the meta-analysis by Román-Caballero et al. (2022) showed that among studies without random assignment of participants, there was a positive and significant baseline difference in favor of musical training groups, indicating that students who voluntarily chose musical training as an extracurricular activity had better initial cognitive and academic scores compared to their peers. This highlights the importance of taking intelligence into account when talking about the relation between mathematics and music.

Additionally, most studies do not distinguish between innate musical potential (or musical aptitude) and musical training that helps to develop manifest musical abilities (Müllensiefen et al., 2022). One study that distinguishes between musical aptitude and musical training was conducted by Swaminathan et al. (2017). While not focusing on mathematics but on general cognitive abilities, the authors were able to show that musical aptitude, defined as the ability to perceive, remember, and discriminate sequences of tones or beats, was associated with intelligence even when musical training was held constant. In contrast, there was no association between musical training and intelligence after controlling for musical aptitude. This finding suggests that rather than studying the effect of musical training on mathematics, the focus should be on musical ability. However, the few studies that have taken a correlational approach between musical and mathematical abilities suffer from several limitations. For example, Manginas et al. (2018) used a rather small sample size of only 36 second-grade children. Using a measure of music listening ability consisting of a tonal and a rhythmic task and a curriculum-based math assessment, they found a strong positive (*r* = 0.70) relationship between the two variables. While using a larger sample size (*n* = 86), Hinojosa (2020) measured music listening ability and mathematics achievement using a state standardized test of eighth-grade band students. However, music listening abilities did not predict mathematics achievement. In addition, neither study included a measure of general cognitive ability. Furthermore, Hinojosa (2020) only included individuals with musical training, and while good musical skills are typically assumed to be a result of musical training, this is not necessarily the case, in particular when only listening skills are considered. Correia et al. (2022) assessed the musical abilities of 190 individuals who had no formal music training and found a wide range of abilities, with some participants having even better musical abilities than the average formally musically trained individual. Similar to the findings of Swaminathan et al. (2017), domain-general cognitive abilities were positively related to musical abilities (*r* = 0.33; Correia et al., 2022). Individual differences in domain-general cognitive abilities are generally also highly correlated with individual differences in mathematical ability and achievement (e.g., Breit et al., 2025).

In sum, the evidence on the relationship between music and mathematics is mixed. This is despite the fact that it can be argued that music and mathematics draw on related representations and similar skills. For example, in both learning contexts, students will encounter reasoning about ordinal systems (e.g., the count list, the ordering of musical pitches). What we can be sure of, however, is that domain-general cognitive abilities cannot be ignored when studying both music and mathematics. Intelligence is reported as the strongest predictor for mathematical achievement (e.g., Breit et al., 2025) and has also been found to be a predictor of musical abilities (e.g., Swaminathan et al., 2017). Thus, the main goal of this study was to investigate the relationship between different facets of musical and mathematical abilities, taking into account general cognitive abilities. This was done in a diverse sample of adults with a wide range of both mathematical and musical abilities. Musical abilities were assessed using three different listening tests measuring beat alignment, mistuning perception, and melodic discrimination abilities; mathematical abilities were assessed using basic numerical abilities, arithmetic fluency, and higher mathematical knowledge; and general cognitive abilities were assessed using a verbal, numerical, and figural subscale of an established intelligence test. We expected small to moderate positive relationships between musical and mathematical abilities (Hypothesis 1), while having no specific assumptions on how these relationships vary for the different facets. In addition, we assumed that musical and mathematical abilities are both positively related to intelligence (Hypothesis 2). Finally, we expected to find a general factor of mathematical abilities and a general factor of musical abilities that explain a substantial amount of variance in the scores of the individual tests (Hypothesis 3a). We expected a positive relationship between these two latent factors (Hypothesis 3b). Furthermore, we assumed that the relationship between the latent factors of mathematics and music decreases when controlling for individual differences in intelligence (Hypothesis 4).

## 2. Materials and Methods

### 2.1. Participants

The final sample consisted of 170^1^ adults (99 female, 68 male, and 2 other^2^; 18–39 years old; *M* = 25.36, *SD* = 4.76). In order to ensure a wide range of both mathematical and musical abilities, the recruitment process focused on three target groups: The first was a mathematics group, which consisted of students or graduates who were working in a field of mathematics, physics, an engineering subject, or a related field. The second was a music group, which was defined as students or graduates of music, music education, musicology, or a related field. The third target group was a designated control group with participants who neither excelled in mathematics nor in music and who were studying or working in a field unrelated to music or mathematics, mostly psychology. However, we had about 10% of individuals who could be classified as belonging to more than one group (e.g., individuals studying mathematics and playing semi-professionally in a band for ten years), and the groups were unequally sized (43 math, 34 music, 73 control, 20 mixed). Note that the study was not set up as a group comparison design, but the recruitment strategy of targeting three different groups of participants aimed at increasing the sample variance on mathematics- and music-related tasks. All main analyses ignored the recruitment group. In other words, all participants, including the mixed ones, were included, and continuous measures of music, mathematics, and general cognitive ability were used. Participants were recruited and tested in both Austria (*N* = 137) and the UK (*N* = 33) and thus tested in either German or English, but we did not expect language to have any effect on performance on the mathematical and music tasks, as they were largely language-independent. Participation was voluntary, all participants gave informed consent, and the study was approved by the local ethics committee. Participants in Austria who were defined as belonging to the math and music group received financial compensation of €22 at the end of the study. Psychology students in Austria were offered a certificate of participation for course credit. In the UK sample, participants either volunteered or received £20 as compensation.

### 2.2. Material

#### 2.2.1. Musical Abilities

Musical abilities were assessed with three tasks focusing on musical perception, which have been shown to load on a single factor (Müllensiefen et al., 2022). The three tasks were hosted on the online testing server of the German Society for Music Psychology (DGM, https://testing.musikpsychologie.de/dots_home/ (accessed on 30 September 2025)) and presented on a computer in the lab using Sennheiser PUNEX KOH2750 headphones, with participants able to adjust the volume. All three tasks were adaptive and scored automatically by the DGM, resulting in a downloadable data file. Scores for each task ranged from minus four to plus four, with higher scores indicating better musical test performance.

##### Computerized Adaptive Beat Alignment Test (BAT)

The BAT (Harrison & Müllensiefen, 2018) measures the ability to derive the underlying beats from pieces of music. Participants listen to two versions of a short musical excerpt (jazz, rock, orchestral pop), both overlaid with a metronome-like beep track. In one version, the beep track does not align with the inherent musical beat, while in the other version musical beat and beep track are perfectly matched in time. Participants have to identify the off-beat version in 25 items, which are selected from a large item pool. Sequential item selection follows an adaptive procedure that is based on an explanatory item response model. It took participants approximately 7 min to complete the BAT. The test has good test–retest reliability (*r_tt_* = 0.67).

##### Mistuning Perception Test (MPT)

The MPT (Larrouy-Maestri et al., 2019) measures the ability to recognize whether the pitch of the singing voice in a short musical excerpt is in tune with the corresponding backing track or not. Participants listen to two versions of a short excerpt from unfamiliar pop music recordings. In one version, the pitch of the singing voice is either shifted higher or lower than the instrumental backing track, while in the other version, the pitch is in tune with the backing track. Participants have to identify the out-of-tune version in 25 items, with item selection again following an efficient adaptive procedure. Participants needed approximately 7 min to complete the task. The test has good test–retest reliability (*r_tt_* = 0.70).

##### Melodic Discrimination Test (MDT)

The MDT (Harrison et al., 2017) measures the ability to discriminate between melodies that differ only slightly. Participants listen to three transposed versions of the same melody. In one version, a single note has been changed, and participants have to identify the melody that differs from the other two, i.e., making it an odd-one-out task paradigm. Participants must solve 20 items, which are drawn from a large item pool based on their performance on the previous item, making it an adaptive test. The MDT took participants approximately 7 min to complete. The test has good test–retest reliability (*r_tt_* = 0.67).

#### 2.2.2. Mathematical Abilities

Mathematical abilities were assessed via three tasks with ascending complexity. While basic numerical abilities only require understanding the concepts of cardinality and ordinality as well as the number symbols one to ten, arithmetic fluency requires the knowledge of arithmetic facts and procedures typically acquired in primary school. The last task focuses on higher mathematical knowledge typically acquired in secondary school.

##### Basic Numerical Abilities (BNA)

The basic numerical abilities task includes three subcomponents: approximate number system, cardinality, and ordinality. All three subcomponents were measured in one computer-based task programmed in PsychoPy (Version 2023.2.3, Peirce et al., 2022). To measure the approximate number system, that is, the nonverbal ability to quantify and approximate the number of objects, we used a non-symbolic numerical comparison task (after Odic & Starr, 2018). Two dot arrays were presented side by side on a computer screen, and participants had to identify which array (left or right) contained more dots without counting. Participants took approximately 5 min to complete 128 trials.

The cardinality task measures the ability to efficiently discriminate and compare symbolic numerical magnitudes (i.e., digits). Two single-digit numbers (1 to 9) with a numerical distance of 1, 2, 5, or 6 were presented side by side on a computer screen. Participants had to judge which digit was the larger one (adapted from Moyer & Landauer, 1967). Participants took approximately 5 min to complete 80 trials.

The ordinality task measures the efficient processing of numerical ordinal knowledge. Participants had to judge whether three single-digit numbers presented on a computer screen were ordered in an ascending sequence (e.g., 2-3-4) or not (e.g., 4-2-3; after Vogel et al., 2021). Participants took approximately 10 min to complete 180 trials.

All BNA tasks were scored for response time and accuracy, and all tasks have good split-half reliability (0.58–0.99, Meier et al., 2024). If participants had an accuracy score below 50% on one of those three tasks, their scores on this specific task were excluded from further analyses because this would indicate below-chance responses, and the mean accuracy for this type of task is approximately 90% in healthy adults (Meier et al., 2024). In addition, mean response times below 200 ms were excluded from analyses, as this is too fast for participants to have actually processed the stimuli.

For further analyses, we averaged the scores of all three tasks for response time and accuracy, as they represent the same construct and correlate positively with each other (see full correlation matrix in Table A1 in Appendix A). Subsequently, we calculated an inverse efficiency score (IES = RT/ACC; see Bruyer & Brysbaert, 2011) to only have one combined score for the basic numerical abilities for all further analyses. As the IES is mostly driven by response time, smaller scores indicate better performance. Since in the other two mathematical tasks, smaller scores indicate worse performance, we reverse-coded the IES score so that higher scores indicate better performance.

##### Arithmetic Fluency

Arithmetic fluency was measured using two subtasks of a paper–pencil arithmetic competency test (Vogel et al., 2017). The first subtask contained 128 simple one-digit multiplications, which are typically solved by retrieving the answer from long-term memory (Hinault & Lemaire, 2016). Participants were given 90 s to solve as many problems as possible. The other subtask comprised 60 complex subtractions with two two-digit operands, which are typically solved using a calculation procedure (Hinault & Lemaire, 2016). Participants were given 120 s to solve as many problems as possible. The number of correctly solved problems was counted per subtask. As the two scores were highly correlated (*r* = 0.67, *p* < .001), they were added to create an overall arithmetic fluency score (0–188).

##### Higher Mathematical Knowledge (MPA)

The official short version of the Mathematics Test for Personnel Selection (MPA, Jasper & Wagener, 2013) was used to measure mathematical competence. This paper–pencil test consists of 31 mathematical problems covering topics such as fractions, unit conversion, exponentiation, division with decimals, algebra, geometry, roots, and logarithms. Participants have a maximum of 15 min to answer these open-ended and multiple-choice questions. The mathematical competence score is calculated by summing the correct answers given, resulting in a raw score ranging from 0 to 31. The short version of the test was found to have a high correlation with the long version (*r* = 0.93) and a high reliability (Cronbach’s α = 0.89; Jasper & Wagener, 2013).

#### 2.2.3. Background and Training Variables

Experience and training are among the most important aspects for performance and expertise (Bilalić, 2017; Gobet, 2015); thus, we used two questionnaires to assess participants’ prior experience, training, and activities with music and mathematics in more detail. For the domain of music, we used the Goldsmiths Musical Sophistication Index (Müllensiefen et al., 2014) as an established inventory of musical experience, training, and skills. As no comparable questionnaire exists for mathematics, we adapted the questions from the Goldsmiths Musical Sophistication Index for the domain of mathematics to create the Mathematics Sophistication Index.

##### Goldsmiths Musical Sophistication Index (Music SI)

The Gold MSI (Müllensiefen et al., 2014; German version by Schaal et al., 2014) is a self-report questionnaire that collects information on general musical activity. The questionnaire includes five subscales, namely active exposure to music, musical education, emotions related to music, singing skills, and musical perception skills. Responses are given on a 7-point Likert scale. A 29-item short version of the questionnaire was administered online via Limesurvey and took approximately 7 min to complete. The Gold MSI shows both a high internal consistency (Cronbach’s α = 0.91 from the German version) and good retest reliability (*r_tt_* = 0.97 from the English version). Additionally, the Gold MSI provides a general musical sophistication factor, which incorporates items from all five sub-scales. The mean score of general musical sophistication, as well as all subscales, was calculated using the Gold MSI Scoring app (https://shiny.gold-msi.org/gmsiscorer/ (accessed on 30 September 2025)). Only the general musical sophistication score was used in the data analyses.

##### Mathematical Sophistication Index (Math SI)

The Math SI was designed to provide a measure of general mathematical experience in order to understand participants’ previous mathematical education history and how much they actively engaged in mathematical tasks in their everyday lives. In addition, the Math SI should be comparable to the Musical SI, so we adapted Goldsmith’s Musical Sophistication questionnaire for the domain of mathematics. Specifically, the musical training and active engagement subscales were adapted by rephrasing the items (e.g., “I spend a lot of my free time doing music-related activities” became “I spend a lot of my free time doing math-related activities”). One item of the active engagement scale (“I do not spend a lot of money on music”) was not used for the Math SI because it did not make sense to translate it to the domain of mathematics. In addition, we included further items from well-used math-specific attitude questionnaires (e.g., “Mathematics is personally important to me” was used for the active engagement subscale (from Trautwein et al., 2006)). This resulted in a final pool of 25 items, all of which were in a 7-point Likert scale format. A full list of all items can be found in the OSF project (https://osf.io/xj9sw/overview?view_only=1569c58c39424070a991e847661514d7 (accessed on 30 September 2025)). For further analyses, we used a general mathematical sophistication index computed by averaging all items^3^. This general score showed good internal consistency (Cronbach’s α = 0.95).

##### Intelligence

As we were interested in the relation between mathematical and musical abilities beyond what can be explained by their shared relation with intelligence, participants completed three tasks from the Intelligence Structure Test (I-S-T 2000 R, Liepmann et al., 2007) in paper–pencil format. This test is based on the hierarchical proto-model of intelligence structure research, a flexible, theory-guided template, which assumes a general factor at the top and broad group factors beneath. More specifically, the three tasks we used were constructed for the assessment of content-related reasoning ability (verbal, numerical, figural) and overall reasoning. In the verbal task (similarities), six words were presented, but only two of them could be described by a common generic term, and participants had to indicate which two they were. In the numerical task (number series), participants had to continue a series of seven numbers. In the figural task (cubes), participants were presented with five different three-dimensional cubes with different patterns on each side. Each item showed one of the five cubes in a different position, and participants had to figure out which one it was. This was done by mentally rotating the cubes. Each task had 20 items of increasing difficulty, and participants had 7 (verbal), 10 (numerical), and 9 (figural) minutes to solve these items. The reliability of the tasks is good (Cronbach’s α = 0.76, 0.91, 0.80). For further analysis, we use raw scores of each subscale by summing all correct answers given and also compute a general reasoning score by averaging the three subscale scores.

### 2.3. Procedure

The study was conducted both online and in person, either individually or in groups with a maximum of 4 participants. In the online part, which was conducted in advance, participants received all information about the study and gave their informed consent. They then generated their personal pseudo-anonymized code, completed the demographic data, and answered both the Math SI and the Music SI questionnaires. This took a maximum of 15 min, after which participants were directed to a link to book a time and date for the in-person portion of the study. The face-to-face part was divided into two test blocks separated by a five-minute break, and approximately half of the participants started with test block one (version 1), while the other half started with test block two (version 2). In version 1, participants began with a creativity task (25 min, results not reported in this study), followed by the three tasks measuring mathematical abilities (35 min). Thus, the first block of testing lasted approximately 60 min, after which participants took a short break. Block two began with the intelligence test (30 min), followed by the musical ability task (25 min), taking roughly 55 min. The total duration of the face-to-face testing session was approximately 120 min.

## 3. Results

All analyses were performed using R Statistical Software (Version 4.5.0) via RStudio (version 2024.12.0), and the full analysis code, as well as a code book with all the used variables, can be found in the OSF project (https://osf.io/xj9sw/overview?view_only=1569c58c39424070a991e847661514d7 (accessed on 30 September 2025)).

### 3.1. Correlation Between Mathematical and Musical Abilities

To test Hypothesis 1, where we expected small to moderate positive relationships between musical and mathematical abilities, we examined Pearson’s correlations between the tasks separately. The correlations in Table 1 show a differentiated pattern. First, basic numerical abilities correlate positively with all three musical tasks, indicating that better basic numerical abilities are associated with better musical abilities. Second, the MDT is significantly correlated with all three mathematical abilities. In contrast, the BAT and MPT correlate significantly only with one or two of the mathematical abilities, and higher mathematical knowledge only correlates with one of the musical tasks.

### 3.2. Correlation Between Intelligence and Mathematical and Musical Abilities

As one of our main interests was how mathematical and musical abilities are related beyond intelligence, we first looked at whether both mathematics and music were positively related to intelligence on their own, as we assumed in Hypothesis 2. As can be seen in Table 2, intelligence correlated positively with all mathematical abilities. Significant correlations were found for all three intelligence subscales as well as for the mean general intelligence score. Numerical intelligence showed the highest correlations with mathematical abilities. In contrast, the correlations between musical abilities and intelligence were weaker than the correlations with mathematical abilities. While both the MPT and the MDT correlated with all the intelligence subscales and showed a moderate correlation with the general intelligence score, the BAT did not correlate significantly with any of the intelligence measures.

### 3.3. Latent Variable Model of the Mathematics–Music Relationship

As we expected that a general factor of mathematical abilities and a general factor of musical abilities were driving the correlations within each set of measures of mathematics and music abilities (for the full correlation matrix showing these associations, see Table A1), we conducted a confirmatory factor analysis (CFA) via the lavaan package in R (Version 0.6.18; Rosseel, 2012). We assumed that BNA, Arithmetic, and MPA load onto mathematical abilities, and BAT, MPT, and MDT load onto musical abilities (Hypothesis 3a), and that there is a positive relationship between the latent variables music and mathematics (Hypothesis 3b). We used maximum likelihood estimation with robust (Huber-White) standard errors and a scaled test statistic that is (asymptotically) equal to the Yuan-Bentler test statistic (MLR) as an estimator; missing data was taken into account using the full information maximum likelihood (FIML) approach.

**Figure 1:**
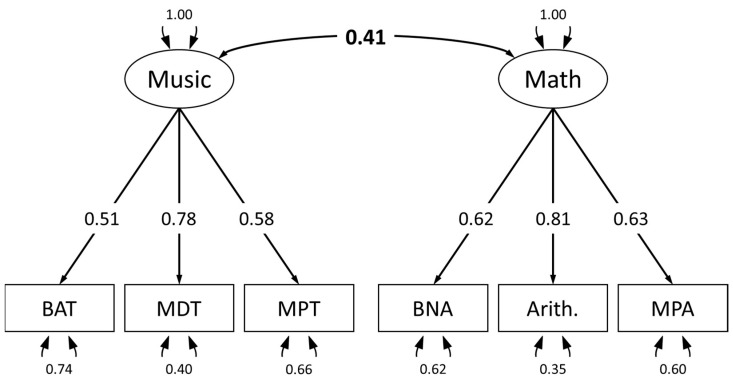
Theoretical assumed model of mathematical (BNA = Basic Numerical Abilities, Arith. = Arithmetic Fluency, MPA = Higher mathematical knowledge) and musical abilities (BAT = Beat Alignment Test, MPT = Mistuning Perception Test, MDT = Melodic Discrimination Test) with standardized loadings derived from CFA.

The assumed model (see Figure 1) showed good model fit. The χ^2^ test yielded a non-significant result (χ^2^ = 10.57; *df* = 8; *p* = .227), and the chi-squared (χ^2^) to degrees of freedom (*df*) ratio was 1.32, with χ^2^/*df* < 3 indicating good model fit. According to Schreiber et al. (2006), RMSEA < 0.08, CFI > 0.95, SRMR < 0.06, and TLI > 0.95 indicate a good model fit, and those recommendations were met by our model (RMSEA = 0.044; SRMR = 0.033; TLI = 0.974; CFI = 0.986), confirming Hypothesis 3a. The two latent variables, mathematics and music, correlated positively with a medium effect size (*r* = 0.41). confirming our Hypothesis 3b.

### 3.4. Latent Variable Model of the Mathematics–Music Relationship Controlling for Intelligence

**Figure 2:**
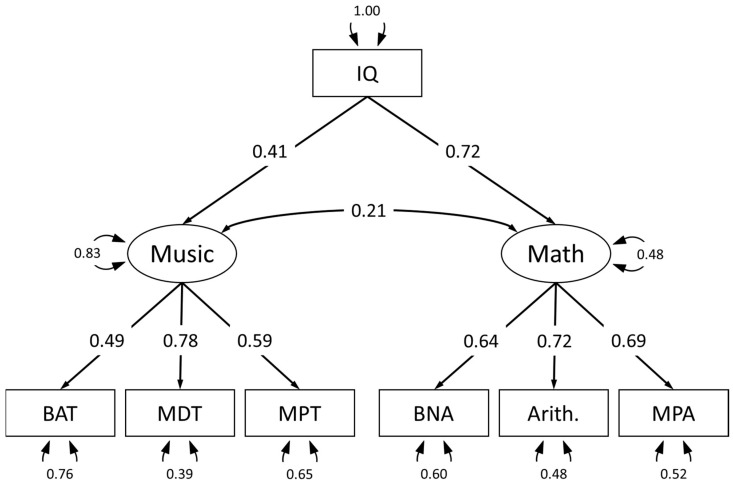
Latent variable model of mathematics (BNA = Basic Numerical Abilities, Arith. = Arithmetic Fluency, MPA = Higher mathematical knowledge) and music (BAT = Beat Alignment Test, MPT = Mistuning Perception Test, MDT = Melodic Discrimination Test) relationship accounting for intelligence (IQ) as a possible common cause.

To account for intelligence as a common cause for the relationship between mathematics and music, we calculated a latent variable model (using unit variance scaling), including general intelligence as a predictor of mathematical and musical abilities, expecting to see a reduced relationship (Hypothesis 4). The overall model fit is good. The χ^2^ test yielded a non-significant result (χ^2^ = 17.48; *df* = 12; *p* = .132), and the chi-squared (χ^2^) to degrees of freedom (*df*) ratio was 1.46, with χ^2^/*df* < 3 indicating good model fit. The recommendations for a good model fit according to Schreiber et al. (2006) were met by our model (RMSEA = 0.052; SRMR = 0.033; TLI = 0.964; CFI = 0.980). General intelligence significantly predicted both mathematical abilities and musical abilities; however, the relation strongly differed. Specifically, the proportion of variance (*R*^2^) that intelligence explains for the latent variable music was relatively low (*R*^2^ = 0.17) compared to the proportion of variance explained in mathematical abilities (*R*^2^ = 0.57; see Figure 2). After accounting for general intelligence in the model, the correlation between mathematics and music is much smaller and also non-significant (*r* = 0.21, *p* = .081). This indicates that intelligence indeed is one of the underlying factors driving the relationship between mathematics and music.

### 3.5. Exploratory Analysis: Regressing Musical Ability on Mathematical Ability and Vice Versa

In a last and exploratory step, we were interested in whether musical abilities can explain a unique amount of variance in mathematical abilities, and whether mathematical abilities can explain a unique amount of variance in musical abilities. As not only general cognitive abilities but also experience and training, as well as affective factors like motivation and interest, are important aspects for performance and expertise (Bilalić, 2017; Gobet, 2015; Breit et al., 2025; Swaminathan & Schellenberg, 2018), both general intelligence and the sophistication indices were included as predictors. First, we calculated the latent factor scores of mathematical and musical ability using the regression method. Then, we used those factor scores in two multiple linear hierarchical regressions. In a first step, we included general intelligence and the respective sophistication indices as predictors. In the second step, we added the latent factor score of the other ability.

As can be seen in Table 3, both general intelligence and mathematical sophistication were significant predictors of mathematical abilities, explaining 42% of the variance. However, when adding musical abilities, the model performed significantly better. Roughly 2% additional variance was explained, and musical abilities were also a significant predictor of mathematical abilities. This indicates that even though the latent variable model in Figure 2 showed that the relation between mathematical and musical abilities is significantly reduced when controlling for intelligence, there still seems to be a unique contribution that connects the two abilities beyond domain sophistication and general intelligence.

This is also supported by the regression using musical ability as the dependent variable. As can be seen in Table 4, both general intelligence and musical sophistication were significant predictors for musical ability, explaining 47% of the variance. However, when adding mathematical ability as a predictor, an additional 4% of the variance was explained. Mathematical ability was also a significant predictor, strengthening the assumption of a unique link between musical and mathematical abilities beyond domain sophistication and general intelligence.

## 4. Discussion

The present study examined the relationship between general mathematical and musical abilities, as well as their specific subcomponents. To answer the question of what kind of shared cognitive architecture underlies a potential relationship between mathematical and musical cognition, we included intelligence as an additional variable in our model. Using a sample of 170 young adults with backgrounds in mathematics, music and other domains, we found that individual mathematical and musical abilities were positively related. Using confirmatory factor analysis (CFA), we found a moderate to strong association between the two latent constructs of mathematical and musical abilities. This suggests that people with better mathematical skills tend to have better musical skills, and vice versa. However, after including intelligence in our model, this relationship became nonsignificant. This finding suggests that intelligence may be one of the driving forces, though not the only one, behind the link between mathematics and music.

Our first hypothesis, which expected small to moderate positive relationships between musical and mathematical abilities, was largely confirmed. However, when considering the individual tasks in the two domains, the picture that emerged was nuanced. Two results especially stood out. First, basic numerical abilities were consistently correlated with all three musical tasks. One possible explanation is that BNA, of all the mathematical tasks, had the most similar task demands and required a similar level of knowledge as the musical tasks. All three musical tasks had a similar modality. Participants had to choose between two or three pieces of music and indicate which piece was off-beat, out of tune, or had a changed melody. Furthermore, none of the musical tasks necessarily require skills linked to learning an instrument or taking formal music lessons (Müllensiefen et al., 2022). Thus, they require very little actual knowledge. The same holds true for the BNA. In the BNA tasks, participants had to choose between two options and indicate whether one dot array had more dots than another, whether one number was larger than another, and whether three digits were ordered or not. Out of all three mathematical tasks, the BNA tasks require the least knowledge. While the ANS task can be solved without formal knowledge—as evidenced by the fact that infants and animals have an approximate number system (Odic & Starr, 2018)—the knowledge required for the cardinality and ordinality tasks is acquired in early primary school or before (Geary et al., 2019). To solve these tasks successfully, individuals must recognize and understand that symbolic numbers represent specific quantities and are part of ordered sequences (Lyons et al., 2016).

Second, another pattern that emerged was that the Melodic Discrimination Task (MDT) was consistently related to all three mathematical abilities and showed the strongest correlations. This may be due to the task demands of MDT compared to the other two tasks. In the Beat Alignment Task (BAT), participants indicate which piece of music is off-beat. In the Mistuning Perception Task (MPT), they indicate which piece is out of tune. In both cases, it is possible to make this judgment directly while hearing the music. For example, if one piece is on-beat, one can conclude that the other must be off-beat. In contrast, in the MDT, participants hear three pieces of music and must indicate which one was altered. Harrison et al. (2017) identified four primary cognitive processes necessary for successfully solving melodic discrimination tasks: perceptual encoding, memory retention, similarity comparison, and decision-making. Perceptual encoding and decision-making are necessary for both the BAT and MPT, but memory retention and similarity comparison are unique to the MDT. Participants must actively retain the melodies’ cognitive representations in memory. This requires working memory, which is a cognitive system that temporarily holds and manipulates information necessary for complex tasks (Wilhelm et al., 2013). Participants then compare the similarities between these cognitive representations, using the different feature representations formed during perceptual encoding and stored in working memory. Working memory is also a cognitive ability that is strongly related to mathematics, as has been supported by several meta-analyses (Friso-van den Bos et al., 2013; Peng et al., 2016). Moderator analyses (Peng et al., 2016) revealed that word problem solving, which was incorporated into our MPA task, and whole-number calculation, which was incorporated into our arithmetic fluency task, exhibited the strongest correlation with working memory. Thus, the cognitive demands on working memory may explain the association between the MDT and mathematics measures. This is supported by the MDT’s consistent and high correlations with intelligence, as intelligence and working memory are also highly related (Ackerman et al., 2005; Colom et al., 2008).

Regarding our second hypothesis, where we assumed that musical and mathematical abilities are both positively related to intelligence, the findings were very similar to those for the first hypothesis. We found mostly evidence for our proposed hypothesis, but when the separate subtasks were considered, a differentiated picture emerged. As would be expected, numerical intelligence showed stronger associations with the mathematical tasks than verbal and figural intelligence. However, when comparing the strength of the relationships among the three subtasks, they were rather comparable, especially when looking at the general intelligence score. In contrast, the picture was different for the music tasks. As previously mentioned, the MDT showed consistent positive correlations with all intelligence subtasks, which might be due to higher cognitive demands (e.g., working memory). The same was true for the MPT, even though the correlations were slightly lower. In contrast, the BAT did not correlate with any of the three intelligence subtasks, suggesting that it may rely more heavily on perceptual encoding than on complex cognitive processes. However, three things have to be kept in mind regarding the intelligence measure employed in this study. First, although well established, the IST 2000 R does not disentangle specific cognitive components that are used during the test. Therefore, future studies should consider using more narrowly defined cognitive ability constructs, such as working memory and processing speed, and examine how they relate to mathematical and musical abilities and how they might mediate the association between musical and mathematical abilities. The second aspect was that we only used one task from each domain instead of three, due to time constraints. Therefore, our general score provides only a rough estimate of general reasoning ability. Third, in line with the manual, we used an averaged intelligence score, as we deemed it important to give equal weighting to all three domains. However, contemporary intelligence research discusses hierarchical and multidimensional models of cognitive ability; therefore, modeling intelligence as a latent variable can also be considered^4^. Consequently, this choice should be regarded as a pragmatic simplification rather than a comprehensive representation of intelligence. Future studies should aim to capture intelligence more broadly.

We found strong evidence in support of our Hypothesis 3a, where we expected to find a general factor of mathematical abilities and a general factor of musical abilities. The mathematics and music domains both showed coherent factors with high factor loadings, and the initial confirmatory factor analyses (CFAs) had very good fit indices. This was not surprising, especially for music, since we chose the three tasks based on a study that found that there is a general factor of musical abilities similar to the g-factor of intelligence, which can be measured using those tasks (Müllensiefen et al., 2022). Although this study was conducted with adolescents, a similar pattern emerged in our adult sample. All three music subtasks were positively correlated with each other (*r*s = 0.30, 0.36, 0.46; all *p*s < .001, see Table A1) and showed factor loadings around 0.50 or higher. However, as all three tasks had the same response modality, this factor may be construed not only because it reflects a shared theoretical or cognitive construct but also due to the common assessment method. This emphasizes the importance of selecting tasks with different response modalities in future studies. The mathematical factor proved to be almost equally consistent. This may come rather unexpectedly because of the following differences between the tasks. First, unlike the musical tasks, the mathematical tasks have different answer modalities. In the three BNA tasks, participants always had to choose between two options. In contrast, in the arithmetic fluency and mathematical achievement task, participants had to produce answers, which is considered harder than forced-choice tasks because guessing is not possible. Second, compared to the music tasks, where the amount of explicit musical knowledge needed to solve the tasks was comparable and minimal in all cases, this differed for mathematics. While the BNA required limited explicit knowledge, the arithmetic fluency task required knowledge typically learned in primary school, and the MPA required knowledge learned in secondary school. According to Gilmore (2023), who proposed a multi-level framework of mathematical cognition, our three tasks would load on different levels. Despite this, all components of the mathematics factor were highly correlated (*r* = 0.35, 0.50, 0.52, all *p* < .001) and showed factor loadings of 0.60 or higher.

We were able to confirm both the second part of the third hypothesis (Hypothesis 3b), where we expected a positive relationship between the two latent factors of math and music, and the fourth hypothesis, where we assumed that the strength of this relationship would decrease when controlling for individual differences in intelligence. The latent factor mathematics and the latent factor music showed a medium-sized positive correlation (*r* = 0.41). This is in contrast to both previous studies that took a correlational approach to investigating the relation between mathematical and musical abilities. Manginas et al. (2018) found a much stronger correlation between a curriculum-based math assessment and a tonal (*r* = 0.63) and a rhythmic task (*r* = 0.66), whereas Hinojosa (2020) did not find a significant relation between a standardized mathematical achievement test and a tonal (β = 0.14) and a rhythmic task (β = −0.11). However, in contrast to our adult sample, both studies used children as participants, and they both used a knowledge-based test of mathematical achievement, whereas our study tried to assess mathematical abilities more broadly. However, although we selected tasks to evaluate musical and mathematical abilities based on prior research and theoretical considerations, these tasks only capture part of the entire construct. Future studies could assess not only basic abilities but also more complex musical and mathematical abilities to obtain a more complete picture. However, this approach may result in findings that are more heavily influenced by experience and acquired knowledge rather than basic and possibly innate ability differences.

Although the difference in fit indices, AIC, and BIC between the models with and without intelligence as a predictor was not substantial, the model with intelligence as a predictor turned out to better represent the data structure, indicating that including some measure of cognitive ability is essential when discussing the relationship between mathematics and music. Further, general intelligence was a significant predictor of both the latent factors of music and mathematics. Our finding that people with higher intelligence scores also perform better in mathematics fits very well with previous literature, with intelligence sometimes being called the most important predictor of mathematical achievement (Breit et al., 2025; Deary et al., 2007). The picture is a little less clear in music. For example, Swaminathan et al. (2017) showed that in a sample of musically trained and untrained adults, music aptitude, defined as the ability to perceive, remember, and discriminate sequences of tones or beats, was positively related to intelligence even when music training was held constant. Some studies also focused on whether individuals with musical training have higher cognitive abilities than those without training. For instance, Schellenberg (2011) demonstrated that 9- to 12-year-olds with musical training had higher IQs than their untrained peers. Criscuolo et al. (2019) examined this issue in adults, comparing musicians, defined as having more than five years of musical training, a degree in music, or receiving monetary compensation for musical performances, with non-musicians who had less than three years of musical training. They found that adults with professional, long-term musical training outperformed non-musicians on standardized cognitive tests designed to measure general intelligence, even after controlling for educational background, socioeconomic status, personality traits, and other demographic variables. However, these results do not indicate whether the observed differences in cognitive abilities are due to pre-existing neurocognitive differences that predispose individuals to engage in musical activities or if musical training influences cognitive abilities. Some researchers argue that music lessons improve attentional and executive functioning, benefiting almost all cognitive tasks (e.g., Hannon & Trainor, 2007).

The exploratory analyses reported in Section 3.5 had two main purposes. First, we wanted to include mathematical and musical sophistication, as experience, training and affective factors such as interest and motivation are predictors for performance in both mathematics and music (Bilalić, 2017; Gobet, 2015; Breit et al., 2025; Swaminathan & Schellenberg, 2018). Indeed, musical sophistication strongly predicted musical ability. Technically, we examined musical abilities that do not require explicit training or any formal musical knowledge; however, extensive musical training improves performance on musical ability tasks, and approximately 40% of our sample had extensive training. Notably, musical sophistication showed higher correlations with musical abilities as the tasks became more cognitive rather than perceptual (see Table A1, BAT: *r* = 0.30, MPT: *r* = 0.40, MDT: *r* = 0.57). In mathematics, the mathematical sophistication index was also a significant predictor of ability. However, while in the domain of music, the sophistication index showed a stronger effect compared to general cognitive abilities; for modeling mathematical ability, general cognitive abilities showed a stronger effect. Nevertheless, mathematical sophistication remained a significant predictor, indicating that mathematical performance is also influenced by affective factors and training. However, as this is a new self-report measure, albeit with very good reliability, we encourage further research on self-report inventories of mathematical background and expertise in the future. Interestingly, we found a pattern similar to that in music. When we examined the bivariate correlations in Table A1, we found that the relationship with self-reported mathematical sophistication became stronger as the tasks became more mathematically demanding (BNA: *r* = 0.13, Arithmetic: *r* = 0.36, MPA: *r* = 0.59). The second main purpose of our exploratory analysis was to determine whether, even after controlling for general cognitive abilities, training, and attitudes towards the domain, there is something unique connecting mathematical and musical abilities. Indeed, musical ability was a significant predictor of mathematical ability, and vice versa, explaining additional unique variance. While the increases were small (two and four percent, respectively), there seems to be something beyond intelligence that connects mathematics and music. As these results conflict to some extent with those from our confirmatory analyses, both findings should be interpreted with caution. Future studies should examine aspects that music and mathematics share, such as representational languages, specific symbolic notations, and fundamental concerns with patterns and structures, to gain a deeper understanding.

One limitation of this study is the sample we used. First, even though we fulfilled various rules of thumb for the required minimal sample size for CFA/SEM (e.g., *N* = 150 as minimum, *N*:*q* (estimated parameters) of 10:1; see Kyriazos, 2018), future studies should aim for a larger sample size and perform an a priori power analysis. In addition to increasing the sample size, future studies should aim for a more diverse sample in terms of general cognitive abilities and education level, as our sample was relatively highly educated. Further, future studies should aim for a language-balanced sample to allow testing whether the relation between mathematics and music holds true in a variety of populations.

In conclusion, when a measure of general cognitive ability, such as intelligence, is included as a predictor of both music and mathematics, the association between the two latent constructs, mathematics and music, decreases significantly and becomes non-significant. However, although intelligence explains most of the shared variance, a small, statistically reliable association between certain musical and mathematical skills remains, consistent with some domain-specific overlap. Therefore, subsequent research should attempt to pin down what drives the unique connection between mathematics and music, and future training studies attempting to foster mathematics through musical training should incorporate and control for measures of cognitive abilities.

## Figures and Tables

**Table 1:** Correlations between mathematical abilities and musical abilities.

	BNA	Arithmetic	MPA
BAT	0.18 *	0.21 **	0.06
MPT	0.19 *	0.13	0.14
MDT	0.24 **	0.21 **	0.27 ***

Notes: BAT = Beat Alignment Test, MPT = Mistuning Perception Test, MDT = Melodic Discrimination Test, BNA = Basic Numerical Abilities, Arithmetic = Arithmetic Fluency, MPA = Higher mathematical knowledge; *** *p* < .001, ** *p* < .010, * *p* < .050; Pairwise deletion was used to handle missing values, *N* = 158 for correlations with BNA, *N* = 168 for all other correlations.

**Table 2:** Correlations between intelligence, mathematical abilities, and musical abilities.

	BNA	Arithmetic	MPA	BAT	MPT	MDT
Verbal Intelligence	0.35 ***	0.18 *	0.38 ***	0.09	0.16 *	0.17 *
Figural intelligence	0.30 ***	0.23 **	0.33 ***	0.13	0.23 **	0.25 ***
Numerical intelligence	0.42 ***	0.58 ***	0.48 ***	0.10	0.21 **	0.29 ***
General intelligence	0.48 ***	0.48 **	0.53 ***	0.14	0.27 ***	0.33 ***

Notes: BNA = Basic Numerical Abilities, Arithmetic = Arithmetic Fluency, MPA = Higher mathematical knowledge, BAT = Beat Alignment Test, MPT = Mistuning Perception Test, MDT = Melodic Discrimination Test; *** *p* < .001, ** *p* < .010, * *p* < .050; Pairwise deletion was used to handle missing values, *N* = 158 for correlations with BNA, *N* = 168 for all other correlations.

**Table 3:** Multiple linear hierarchical regression model for mathematical abilities predicted from the general intelligence, mathematical sophistication index, and musical ability.

	Variables	β	*p*
Mathematical Ability			
Model 1 *Adj. R*^2^ = 0.42 *F*(2, 154) = 57.24, *p* < .001	General intelligence	0.53	<.001
	Mathematical sophistication	0.25	<.001
Model 2 *Adj. R*^2^ = 0.44 *F*(3, 153) = 41.25, *p* < .001 *R*^2^*_Change_* = 0.02, *p* = .018	General intelligence	0.46	<.001
	Mathematical sophistication	0.25	<.001
	Musical ability	0.16	.018

**Table 4:** Multiple linear hierarchical regression model for musical ability predicted from the general intelligence, musical sophistication index, and mathematical ability.

	Variables	β	*p*
Musical Ability			
Model 1 *Adj. R*^2^ = 0.47 *F*(2, 154) = 69.62, *p* < .001	General intelligence	0.41	<.001
	Musical sophistication	0.54	<.001
Model 2 *Adj. R*^2^ = 0.51 *F*(3, 153) = 54.49, *p* < .001 *R*^2^*_Change_* = 0.04, *p* < .001	General intelligence	0.25	<.001
	Musical sophistication	0.56	<.001
	Mathematical ability	0.26	<.001

## Data Availability

All data as well as the analyses can be found at https://osf.io/xj9sw/overview?view_only=1569c58c39424070a991e847661514d7 (accessed on 30 September 2025).

## Notes

1 One individual aged 50 was excluded from the data as most of the tasks in the study included a speed component and processing speed declines throughout life (Verhaeghen, 2013).

2 Descriptive statistics for one individual missing.

3 Two items were excluded when calculating the mean score. For the first item, ‘In total, I have had ____ years of mathematics education in my entire life (school, university, college, etc.)’, roughly 15% of respondents provided unrealistic answers, stating fewer than 12 years of formal mathematics education; some even stated zero years. As all participants had completed secondary education, this was not possible. Furthermore, the answer format differed from that of all the other items. The second item was ‘I have received ____ years of additional math instruction/extra hours of math education outside of regular math classes at school (e.g., enrichment programs)’. Here, approximately 75% stated zero years, which made this item extremely skewed.

4 We performed an additional analysis in which intelligence was treated as a latent variable. This can be found on the OSF project (https://osf.io/xj9sw/overview?view_only=1569c58c39424070a991e847661514d7 (accessed on 30 September 2025)). Including intelligence reduced the correlation between mathematical and musical abilities, regardless of whether a mean score or latent variable approach was used.

## Appendix A

**Table A1:** Full correlation matrix.

	BNA	Arithmetic	MPA	Mathematical Sophistication	BAT	MPT	MDT	Musical Sophistication	Verbal Intelligence	Figural Intelligence	Numerical Intelligence
Arithmetic	0.50 ***										
MPA	0.35 ***	0.52 ***									
Mathematical sophistication	0.13	0.36 ***	0.59 ***								
BAT	0.18 *	0.21 **	0.06	−0.13							
MPT	0.19 *	0.13	0.14	0.08	0.30 ***						
MDT	0.24 **	0.21 **	0.27 ***	0.14	0.38 ***	0.46 ***					
Musical sophistication	0.06	−0.01	−0.09	−0.04	0.30 ***	0.40 ***	0.57 ***				
Verbal intelligence	0.35 ***	0.18 *	0.38 ***	0.16 *	0.09	0.16 *	0.17 *	0.05			
Figural intelligence	0.30 ***	0.23 **	0.33 ***	0.25 **	0.13	0.23 **	0.25 ***	0.08	0.23 **		
Numerical intelligence	0.42 ***	0.58 ***	0.48 ***	0.30 ***	0.10	0.21 **	0.29 ***	−0.02	0.40 ***	0.37 ***	
General intelligence	0.48 ***	0.48 ***	0.53 ***	0.33 ***	0.14	0.27 ***	0.33 ***	0.04	0.67 ***	0.72 ***	0.84 ***

Notes: BNA = Basic Numerical Abilities, Arithmetic = Arithmetic Fluency, MPA = Higher mathematical knowledge, BAT = Beat Alignment Test, MPT = Mistuning Perception Test, MDT = Melodic Discrimination Test; Computed correlation used Pearson method with pairwise deletion without correction for multiple comparison; *** *p* < .001, ** *p* < .010, * *p* < .050; Pairwise deletion was used to handle missing values, *N* = 158–168.
